# P081 Thinking beyond the temporal artery: an atypical presentation of giant cell arteritis

**DOI:** 10.1093/rap/rkag113.111

**Published:** 2026-11-03

**Authors:** Spurthi Venkatesh, Siwalik Banerjee, Chamith Rosa

**Affiliations:** University Hospitals of Coventry and Warwickshire NHS Trust, Coventry, United Kingdom; University Hospitals of Coventry and Warwickshire NHS Trust, Coventry, United Kingdom; University hospitals of Coventry and Warwickshire NHS Trust, Coventry, United Kingdom

## Abstract

**Introduction:**

Giant cell arteritis (GCA) is the most common large-vessel vasculitis affecting adults over 50 years of age. It is broadly classified into cranial and large-vessel subtypes, with the superficial temporal artery being the most frequently involved cranial vessel. Typical manifestations include new-onset temporal headache, scalp tenderness, jaw claudication, visual symptoms, and elevated inflammatory markers. However, involvement of other branches of the external carotid artery, such as the occipital artery, is uncommon and may present atypically, posing a diagnostic challenge. We report a case of isolated occipital artery GCA identified on ultrasonography despite negative temporal and axillary artery imaging.

**Case description:**

A 66-year-old woman presented with a three-week history of abrupt-onset left-sided occipital headache, localised to the occipital region without visual or other ocular symptoms. There were no symptoms suggestive of polymyalgia rheumatica or constitutional features.

Her medical history included hypothyroidism, treated with levothyroxine, and chronic obstructive pulmonary disease managed with salbutamol inhalers. Clinical examination demonstrated tenderness over the occipital scalp, while bilateral temporal arteries were palpable and non-tender. Peripheral pulses were normal with no bruits, and musculoskeletal and systemic examinations were otherwise unremarkable.

Laboratory investigations revealed markedly elevated inflammatory markers (ESR 70 mm/hr, CRP 109 mg/L), with all other blood tests within normal limits. WBC was normal, no focus of infection–no other explanation.

Doppler ultrasound of the temporal and axillary arteries showed no evidence of arterial inflammation. However, targeted ultrasound of the occipital artery demonstrated echolucent wall thickening of the right occipital artery, consistent with giant cell arteritis (GCA).

The patient was commenced on high-dose corticosteroid therapy, resulting in rapid clinical improvement of her headache and jaw symptoms, with inflammatory markers normalising (CRP reduced to 6 mg/L).

**Discussion:**

Giant cell arteritis (GCA) is the most common idiopathic systemic vasculitis affecting adults over 50 years of age. We report an atypical presentation of cranial GCA with isolated occipital headache and jaw claudication despite negative temporal artery Doppler ultrasonography. The diagnosis was established following targeted ultrasonography of the occipital artery, highlighting the importance of investigating symptomatic vessels when clinical suspicion remains high.

Cranial GCA most commonly affects the superficial temporal arteries, presenting with temporal headache, scalp tenderness, jaw claudication, visual disturbance, and elevated inflammatory markers. However, inflammation may also involve other branches of the external carotid artery, including the occipital, facial, maxillary, and lingual arteries. Isolated occipital artery involvement is uncommon and may result in delayed diagnosis, as current imaging protocols primarily assess the temporal and axillary arteries.

Pinnell et al. described four patients presenting with occipital headache in whom ultrasonography demonstrated characteristic inflammatory changes confined to the occipital arteries despite atypical clinical features. One patient developed irreversible visual loss before diagnosis, underscoring the importance of early recognition. Their literature review concluded that targeted ultrasonography of the occipital artery can identify GCA when temporal artery imaging is normal and recommended imaging of symptomatic vessels in patients with atypical presentations.

These findings are supported by Guggenberger et al., who demonstrated isolated occipital artery inflammation in 14% of treatment-naïve patients with GCA using high-resolution MRI. The authors highlighted that occipital artery involvement may be overlooked because the vessel is not routinely examined and is technically more challenging to assess with ultrasonography due to its anatomical course.

Our case reinforces that a negative temporal artery ultrasound should not exclude GCA when clinical suspicion remains high. In patients presenting with occipital headache, jaw claudication, and elevated inflammatory markers, targeted imaging of the occipital artery should be considered.

**Key learning points:**

• GCA classically affects the superficial temporal arteries and presents with temporal headache, scalp tenderness, jaw claudication, visual symptoms, and elevated inflammatory markers. However, inflammation may also involve other branches of the external carotid artery, including the occipital, facial, maxillary, and lingual arteries.

• Targeted imaging of symptomatic cranial vessels, including the occipital artery, should be considered in patients with atypical presentations, as disease may be confined to less commonly affected branches of the external carotid artery.

• The occipital artery is not routinely assessed during standard GCA ultrasonography and can be technically challenging to evaluate because of its deep anatomical course and operator dependence, potentially contributing to missed diagnoses.

